# Quantitative Single-Cell Multielemental Analysis Using Metal-Loaded Microparticles: toward a Universal Quantification Workflow in Mass Cytometry

**DOI:** 10.1021/acs.analchem.6c02340

**Published:** 2026-07-24

**Authors:** Ángela de la Rosa-Díaz, Carlos López-Portugués, Mario Corte-Rodríguez, María Montes-Bayón

**Affiliations:** † Department of Physical and Analytical Chemistry, 16763 University of Oviedo. Julián Clavería 8, 33006 Oviedo, Spain; ‡ Health Research Institute of Asturias (ISPA). Av. De Roma s/n, 33011 Oviedo, Spain

## Abstract

The use of lanthanide-tagged antibodies enables multiplexed biomarker monitoring in individual cells by mass cytometry. Here, we show the design and application of a quantitative workflow that enables multiple-element determination in single-cell events using multielement barcoded polystyrene beads and mass cytometry. By online mixing of the cell suspension with the beads, transient events of both entities can be discriminated in the time-of-flight–mass spectrometry (TOF–MS). The element-specific content of the beads is used as a single-point calibration standard to assess the concentration of the same element used as a tag on labeled cells. Using this approach, the quantification of Lu in lymphocytic leukemia cells (MEC-I) labeled with a Lu-tagged anti-CD20 antibody is demonstrated. Moreover, multielemental analysis is shown by obtaining a polynomial model that relates the instrumental response for bead events to the *m*/*z* of the multiple elements and isotopes, enabling quantification of elements not included in the beads. This provides a general workflow for all lanthanides potentially used as tags and is tested in A2780 ovarian cancer cells labeled with Nd-tagged antitransferrin receptor 1 antibody and an Ir DNA intercalator. Excellent agreement with external calibrations is shown, highlighting enhanced quantitative capabilities of high-dimensional single-cell MS, applicable also to single-cell ICP–MS using TOF analyzers.

## Graphical abstract

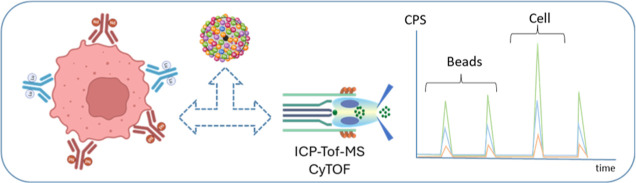

## Introduction

Understanding the biochemical and functional heterogeneity of individual cells is a central challenge in modern biology, immunology, oncology, and clinical translational research. Flow cytometry, and, more recently, spectral flow cytometry,^1^ have become essential tools for high-dimensional single-cell analysis. However, obtaining robust quantitative information at the single-cell level remains challenging, particularly when comparing measurements across experiments and laboratories. Quantitative approaches have been developed for fluorescence flow cytometry, typically relying on calibration beads and standardized antibody conjugates to improve interexperimental comparability.^2–4^ Nevertheless, the accuracy of these methods remains influenced by fluorophore-specific properties and labeling variability, highlighting the need for alternative quantitative single-cell analytical strategies.

Mass cytometry, or cytometry by time-of-flight (CyTOF), which was introduced by the team of Bandura in 2009, has emerged as a technological breakthrough by substituting fluorophores with isotopically pure metal tags, whose signals are detected by TOF elemental mass spectrometry (MS) using an inductively coupled plasma as ion source (ICP–TOF–MS).^5^ This technology has allowed to remove nearly all spectral overlaps and enabled experiments that can routinely include 40–60 parameters, including not only protein biomarkers but also DNA markers and metallodrugs or nanoparticles.^6,7^ Numerous studies have taken advantage of the highly multiplex capabilities of CyTOF to investigate immune responses, characterize tumor-infiltrating lymphocytes, or follow hematopoietic differentiation with greater resolution than was previously possible.^8–10^ Indeed, the technique has become central to many multiparametric immunology pipelines by facilitating deeper functional analyses, from mapping subtle cell-cycle states^11^ to profiling intracellular cytokine signatures and activation markers^12^ that are often difficult to resolve by fluorescence-based approaches.^13^

Beyond its multiplexing capability, an often overlooked advantage of mass cytometry is its potential for elemental quantification. Unlike fluorescence-based approaches, ICP–MS-based detection enables direct conversion of signal intensities into elemental amounts through established calibration strategies. This principle, successfully applied for single-cell elemental analysis by ICP–MS (SC–ICP–MS), has shown to be suitable in different applications. Thus, quantification of intrinsic^14–17^ or exogenous^18–20^ elements, as well as protein biomarkers following metal-labeling strategies^21,22^ compare well with the bulk analyses on the same cell suspension. However, despite the efforts made to develop quantitative strategies, mass cytometry remains as a mainly qualitative strategy, where very few approaches have explored its unquestionable quantification potential.^4,23–25^

CyTOF data quality depends critically on managing instrumental variability. Plasma fluctuations, partial clogging of the sample introduction system or injector and periodic retuning, introduce changes in sensitivity that can obscure biological differences between samples measured at different times or in different instruments. To mitigate these effects, CyTOF applies multielement calibration polymeric beads, such as EQ six-element calibration beads, that are mixed with every sample at a fixed concentration before nebulization.^26,27^ These beads, containing defined quantities of yttrium, indium, cerium, terbium, lutetium and bismuth, allow computational normalization and provide a reference signal that corrects for shifts in sensitivity over time. More recently, approaches using frozen antibody master mixes, unified acquisition templates, and sophisticated normalization algorithms have been introduced to enable reproducible multibatch studies and harmonize data sets across different research centers.^27,28^

Since the sought beads that contain multiple elements in a wide mass range (from ^89^Y to ^209^Bi) are commercially available and show high reproducibility, they could serve as quantitative (single-point) analytical standards (once characterized accordingly). This use would increase their analytical value not only as mere drift correctors but also as calibrants that are present within the sample (matrix-matched). Such a strategy is aligned with recently proposed particle-based calibration approaches in SP/SC–ICP–MS, which exploit transient events of the same nature as the analyte to avoid transport-efficiency-related uncertainties.^29^ In this way, the estimation of parameters like transport efficiencies of the ionic standards is eliminated, reducing the associated uncertainties. Moreover, since the beads are routinely added to all samples in CyTOF, any raw data file could be reprocessed at any time to extract any quantitative information without any additional measurements.

Despite their routine use for signal normalization, CyTOF calibration beads have not been systematically explored as quantitative standards for single-cell elemental analyses. Their use for quantification implies several analytical challenges, including the accurate characterization of their elemental content, the translation of bead-derived responses into cellular elemental amounts, and the extension of the calibration strategy to isotopes that are not present in the beads. Addressing these challenges is essential before calibration beads can be used as reliable quantitative reference.

An interesting additional feature of plasma-based techniques is their relatively uniform sensitivity across the entire mass range. It has been described that the observed instrumental response in most ICP–MS can be fitted to a simple equation as a function of the *m*/*z* of the isotope to be measured for the elements with similar ionization in the plasma, such as the lanthanides.^30,31^ If the transient signals of the multielemental beads are used to obtain the instrumental responses and these are empirically modeled across the monitored *m*/*z* range, a practical route toward multielemental quantification strategy is obtained. This would represent a quantification workflow for multiplex single-cell elemental analysis that repurposes multielement CyTOF calibration beads as generic standards. Such an approach seeks to address the limitations in calibration procedures and, ultimately, to enhance the quantitative applications of high-dimensional single-cell MS that can be applied not only to CyTOF-based analysis but could also be extended to any other SC–ICP–MS analysis using any other TOF mass analyzers.

## Experimental Section

### Instrumentation

The single-particle ICP–MS measurements were performed by using the iCAP TQ triple quadrupole ICP–MS system from Thermo Fisher Scientific (Bremen, Germany). All measurements were done using the standard configuration for sample introduction, including the Micro-Mist nebulizer at a sample flow rate of 0.4 mL min^–1^ and a cyclonic spray chamber. The sample introduction setup was used together with a Teledyne Cetac ASX-560 autosampler. SP-ICP-MS characterizacion of the lanthanide-loaded polystyrene beads was carried out setting a dwell time of 5 ms. Daily optimization of ICP–MS was performed to enhance signal sensitivity, ensuring that the generation of oxide ions and doubly charged species remained below 3% and 5%, respectively.

The CyTOF experiments were conducted in a CyTOF XT (Standard BioTools, San Francisco, CA, USA). This instrument is an ICP–MS that operates TOF mass analyzer with a shortened mass coverage from *m*/*z* 75 to 209. The samples are placed in a refrigerated carousel at 4 °C and injected into the CyTOF with a hydraulic autosampler that is able to handle sample introduction and system washing. The autosampler automatically adds the EQ6 calibration beads (Standard BioTools) to every sample at a 1:10 dilution. After mixing, the bead-containing sample is injected at 30 μL·min^–1^ through a pneumatic nebulizer and a spray chamber that is heated at 200 °C. using a constant sample flow of at 10 μL min^–1^. Microparticle imaging was carried out with a JEOL JEM-2100F transmission electron microscope.

### Labeling of Antibodies Using MAXPAR

Several antibodies were used, including the human anti-ErbB2 receptor antibody (HER2) antibody and the human antitransferrin receptor 1 (TfR1) antibody that were both purchased from R&D Systems (Minneapolis, MN, USA). Both antibodies were carrier-free CyTOF-ready. The human anti-CD20 antibody was kindly provided by the Asturian University Hospital (HUCA) as the pharmaceutical preparation rituximab. All antibodies were labeled using the Maxpar X8 Antibody Labeling Kit (Standard biotools, San Francisco, CA, USA), following the instructions of the manufacturer. The polymer was loaded with ^175^Lu for anti-HER2 and anti-CD20, and with ^142^Nd for anti-TfR1. For the reduction of the antibody, tris­(2-carboxyethyl)­phosphine was purchased from Sigma-Aldrich. For the purification steps, centrifugal filter units of 3 kDa and 50 kDa were used (Amicon Ultra 0.5 mL, Merck Millipore). For cell fixation, a buffered aqueous solution of formaldehyde 4% (v/v) (VWR Chemicals, Pennsylvania, USA) was used. The antibody concentration was determined after the labeling reaction spectrophotometrically using a NanoDrop 2000 (Thermo Fisher Scientific).

The efficiency of the labeling process was evaluated using size exclusion chromatography (SEC) online coupled to ICP–MS as an elemental detector. The chromatographic separation was carried out under isocratic conditions for 45 min using 50 mM ammonium acetate as the mobile phase at a flow rate of 0.7 mL·min^–1^. Both the elemental signal of labeling isotope and the absorbance signal at 280 nm were monitored after the chromatographic separation. Once the labeling and purity of the product were confirmed, the concentration of the corresponding lanthanide was determined by flow injection analysis into the ICP–MS. The number of lanthanide atoms per antibody (stoichiometry) was determined by using the lanthanide and antibody concentrations to calculate the ratio of lanthanide atoms per antibody. This methodology is described in a previous publication.^24^

### Cell Samples

B-cell chronic lymphocytic leukemia MEC-I cell line was grown in Iscove’s modified Dulbecco’s medium (LabClinics, Barcelona, Spain) supplemented with 10% v/v fetal bovine serum (Gibco, Life technologies, Madrid, Spain). The ovarian cancer cell line A2780 was grown in Roswell Park Memorial Institute 1640 Medium (RPMI 1640, Invitrogen, Fisher Scientific, Madrid, Spain) supplemented with 10% v/v fetal bovine serum. Both culture media were supplemented with 1% penicillin–streptomycin. Cells were grown in T-75 flasks at 37 °C in a 5% CO_2_ atmosphere. For cell passaging, A2780 cells were washed three times with PBS and detached using trypsin. MECI cells are nonadherent and were maintained in suspension and passaged by centrifugation and resuspension in fresh medium. The cell number was determined by absolute counting in a Neubauer chamber.

### Labeling of Cells Using MAXPAR Tagged Antibodies and Iridium

Cells were collected by trypsinization, counted using a Neubauer hemocytometer, and adjusted to 3 million cells per aliquot. The cells were then processed following the Maxpar cell surface staining with fresh fix protocol provided by Standard BioTools. Cells were resuspended in Maxpar cell staining buffer and incubated with the metal-tagged antibodies (anti-CD20, anti-TfR1, and/or anti-HER2) for 30 min at room temperature. The concentrations of the antibodies were previously established by proper titration. After staining, cells were washed twice with Maxpar cell staining buffer to remove unbound antibodies.

For cell fixation, cells were resuspended in 1 mL of freshly prepared 1.6% formaldehyde solution and gently vortexed, followed by incubation for 15 min at room temperature.

DNA labeling was subsequently performed by incubating the fixed cells overnight at 4 °C in 1 mL of Maxpar Fix and Perm Buffer supplemented with 125 nM Cell-ID Intercalator-Ir (Standard BioTools). On the following day, cells were washed twice with Maxpar Cell Staining Buffer and twice with Maxpar Cell Acquisition Solution, then resuspended in Cell Acquisition Solution at a final concentration of 0.5 million cells per milliliter. Prior to acquisition, samples were supplemented with EQ Four Element Calibration Beads (Standard BioTools Inc., South San Francisco, CA, USA) at a 1:10 dilution, as specified in the standard CyTOF XT injection protocol.

### Characterization of Lanthanide-Labeled Polystyrene Beads

As normalization and quantification standards, EQ Six Element Calibration Beads from Standard BioTools were used. They are polystyrene microspheres that contain yttrium, indium, cerium, terbium, lutetium, and bismuth. Their size and homogeneity were studied by scanning electron microscopy (SEM) using a JEM-2100F (JEOL, Tokyo, Japan). Single-particle ICP–MS (SP–ICP–MS) was used to quantify the mass of each of the immobilized metals. For this aim, calibration curves of the individual metal solutions were prepared. The transport efficiency (TE) of the elemental standards was estimated using 30 nm gold nanoparticles in the quality control material LGCQC5050 (LGC Standards). For TE calculation, the particle number method^32^ was used. Then, the suspension of the beads was introduced into the SP–ICP–MS and the signal recorded, sequentially, for the elements under evaluation using 5 ms integration time. The mass of each element in the beads was calculated by eq 1, where *m*_p_ is the mass of the element in the particle, η is the TE of the elemental standard solutions, *F* is the sample flow rate, *t* is the integration time (5 ms), *I* is the intensity of the corresponding particle event (only one-point events with 5 ms dwell time), and *b* is the slope of the calibration curve.

1 $$m_{p}=\frac{\eta \cdot F\cdot t\cdot I}{b}$$

A detailed discussion of the obtained elemental mass distributions and their implications for quantitative calibration is provided in the Results and Discussion section.

### External Standard Calibration Quantification in CyTOF

Following the same principles as for SP–ICP–MS calibration, the mass of an element per cell was calculated following eq 1 in CyTOF, as discussed and validated in a previous publication.^24^ The TE was determined using the same gold nanoparticles. In this case, upon adequate dilution, they were set as a sample in the sample carousel and introduced into the CyTOF without the addition of calibration beads. This is because the gold nanoparticles tend to aggregate onto the polymeric beads, causing an underestimation in the determination of the number of gold events. Following the particle number method, the TE for the CyTOF sample introduction system was calculated as eq 2, where *N* is the number of gold events detected, *c* is the gold nanoparticle concentration in the standard in particles per milliliter, *F* is the sample flow rate (standard 30 μL·min^–1^ for CyTOF XT), and *t* is the measurement time (typically 5 min).

2 $$TE=\frac{N}{c\cdot F\cdot t}$$

In the case of former CyTOF versions, the elemental standards can be acquired using the so-called “solution mode” in software. This mode does not exist anymore in recent versions. Therefore, the acquisition of standards in solution must be carried out as a sample and extracting the average intensity from the SPR file that can be transformed into a text file.

### Bead-as-Quantification Standard

The EQ Six Element Calibration Beads were added to all the samples as part of the acquisition workflow in CyTOF XT for signal normalization. In this case, these beads were also used for quantification in the bead-as-quantification standard (BaQS) strategy.

For every sample, standard data cleanup procedures for CyTOF were conducted,^33^ with the modification that the bead events were, in this case, identified and positively selected as a population based on their characteristic multielemental signature. Events meeting only the bead gating criteria (positive to at least two of the contained isotopes) were selected and exported to a microsoft excel worksheet (BaQS). Then, the average intensity of each isotope of the bead events was calculated and divided by the experimentally determined mass of the isotope per bead to obtain a bead-specific response factor. This response factor was subsequently applied to convert integrated cellular signals of the same isotope into an absolute mass. The rationale behind this approach and its analytical performance is discussed in the Results and Discussion section.

### Bead-Inferred Response Model

The bead-inferred response model (BiRM) also used the EQ Six Element Calibration Beads as standards for quantification. In this case, BiRM allowed the estimation of the instrumental response across the monitored mass range using the multielemental composition of the calibration beads and relying only on parameters that were directly measured in the same acquisition as the cell samples (matrix-matched).

The multielement-containing beads were, as in the previous case, normally included in all samples, as recommended by the manufacturer. The average intensity for every isotope contained in the beads and the individual instrumental molar responses were calculated following the same procedure as previously explained for BaQS, dividing the average intensity of each isotope by its average molar content in the beads. The mass-based response factors were converted to molar response factors by application of the atomic mass and natural isotopic abundance of each isotope. These molar response values were then plotted as a function of the mass-to-charge ratio (*m*/*z*) of the corresponding isotopes. An empirical polynomial function of fourth order was fitted to the response versus *m*/*z* data by using least-squares regression. The model validation and limitations are discussed in the Results and Discussion.

The resulting polynomial function defines the BiRM response curve in a specific instrument for a specific sample and was used to estimate the molar response factor for any isotope within the calibrated *m*/*z* interval (89–209). For cell-associated isotopes not present in the beads, the predicted molar response factor was obtained by evaluating the polynomial function at the corresponding *m*/*z* values. Quantification of these isotopes was then performed by dividing the integrated cellular signal by the predicted response factor, yielding the elemental amount (femtograms) per cell. The complete data treatment workflow for BiRM was carried out with an in-house programmed R script that is included as Supporting Information.

A new BiRM response function was generated independently for each data set acquired. Therefore, each sample was quantified with its own BiRM response function.

A simplified workflow diagram showing both the BaQS and BiRM strategies is displayed in Figure 1.

**Figure 1:**
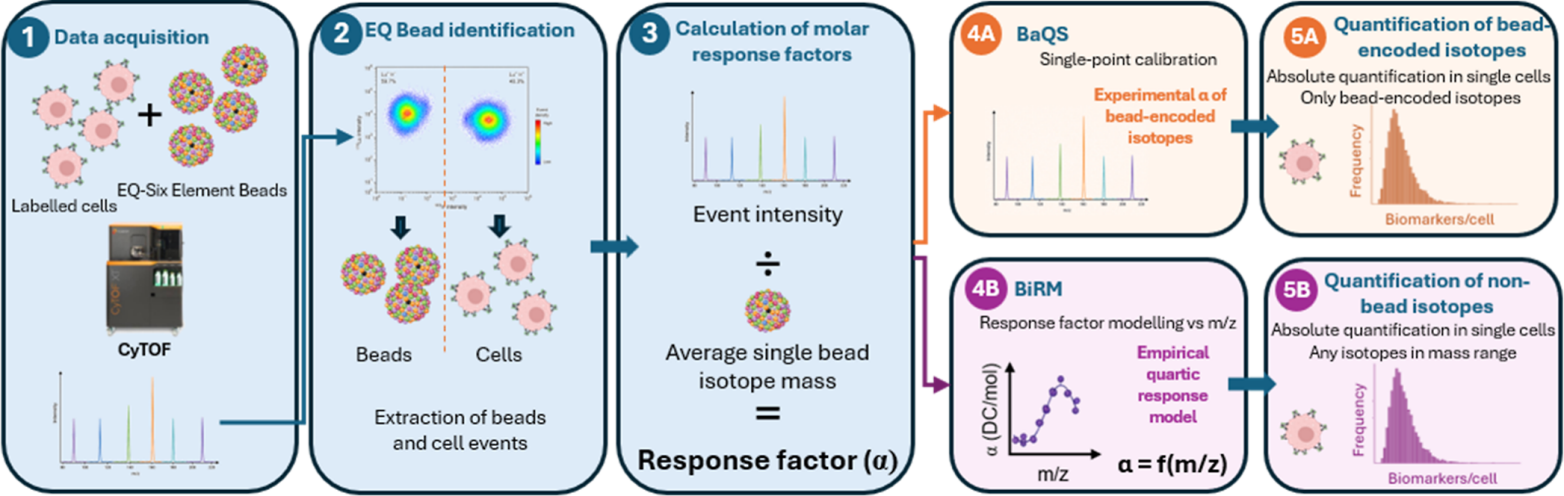
Overall workflow of the proposed BaQS and BiRM strategies for quantitative single-cell elemental analysis by CyTOF and ICP–TOF–MS.

## Results and Discussion

### BaQS: A Single-Point Calibration

CyTOF uses standard polymeric beads containing several lanthanides to normalize intensities and improve comparability of the results. In modern instruments, like CyTOF XT, these beads are automatically mixed with the cell samples in the autosampler and are commercial designated as EQ Six Calibration Beads. Taking advantage of the need to include the multielemental EQ Six Calibration Beads to normalize and gate event intensities in CyTOF, we explored the possibility of using the elements present in these beads to calculate the mass of these same elements in independent cell events. This idea has been coined BaQS.

The EQ Six Calibration Beads are loaded with natural abundance isotopes of yttrium (Y), indium (In), cerium (Ce), terbium (Tb), lutetium (Lu), and bismuth (Bi). Every bead included in each measurement is registered as an individual event comprised by 10–150 consecutive TOF spectra (pushes) for each isotope. The events from the beads and from the cells can be easily differentiated by the elemental composition (this hampers the application of this methodology to quadrupole-based systems). By addressing the individual elemental content in the beads, it should be possible to use them as single-point event calibrants for quantification by calculating the mass response factor (α_i_). This response factor can be calculated with eq 3, where *I*_*i*_ is the average integrated intensity of an isotope *i* in the beads and *m*_*i*_ is the average mass of this isotope in the beads.

3 $$\alpha_{i}=\frac{I_{i}}{m_{i}}$$

The use of bead-derived response factors for single-cell quantification relies on the concept of using particles as calibrants for the quantification of other particles (calibrating entities with entities),^29^ such as cells. In ICP–MS-based techniques, the original chemical form of the analyte is effectively removed through vaporization, atomization, and ionization processes occurring in the plasma. Consequently, provided that complete ionization is achieved, the instrumental response is expected to depend primarily on the elemental mass introduced into the plasma rather than on the nature of the carrier entities. This assumption is particularly reasonable in the present case, as both calibration beads and metal-labeled cells are introduced as micrometer-sized particles, with a carbon-based matrix, and contain lanthanide elements that are efficiently ionized under ICP conditions. Therefore, once the cells or particles reach the plasma, no significant differences in ionization are expected between the two entities. Under this premise, metal-containing calibration polymeric beads and metal-labeled cells can be regarded as analogous transient elemental events, enabling the response factors obtained from the former to be applied to quantitative single-cell analysis.

For the development of BaQS, a previous thorough characterization of the EQ Six beads in terms of element mass per bead had to be carried out. For this aim, SP–ICP–MS using the triple-quadrupole ICP–MS and sequential measurements of the different elements were applied. Experimental Section details on this characterization are included in the Methods section. Quantification was based on using elemental standards for calibration, as previously established, and as detailed in the Experimental Section included in the Methods section. The results of this characterization of the beads are shown in Figure 2. Figure 2A–F shows the elemental mass distribution of the elements present in the beads using the same intensity of the *Y*-axes for comparative purposes.

**Figure 2:**
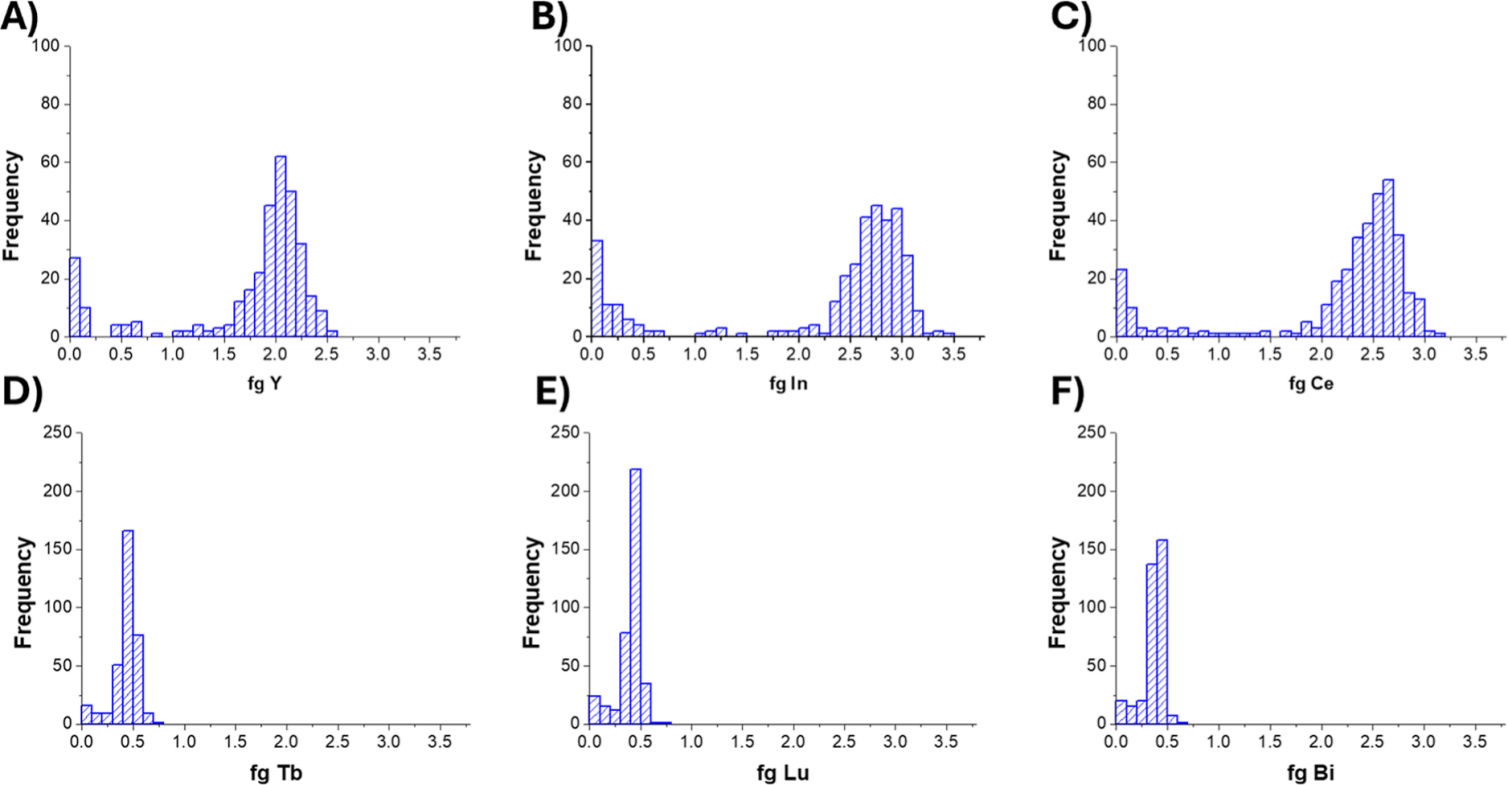
Histograms showing the elemental mass distribution of the beads for (A) yttrium, (B) indium, (C) cerium, (D) terbium, (E) lutetium, and (F) bismuth.

The results of quantitative characterization are summarized in Table 1.

**Table 1:** Results Obtained for the Analysis of the Elemental Content of the Analyzed Beads Using the SP–ICP–MS Analysis Mode

element	Y	In	Ce	Tb	Lu	Bi
average (fg)	2.02	2.76	2.50	0.47	0.44	0.40
SD	0.28	0.26	0.26	0.07	0.06	0.06
RSD (%)	13.69	9.48	10.28	14.54	13.40	15.30
median	2.04	2.77	2.54	0.47	0.44	0.40
Q1	1.91	2.61	2.33	0.42	0.40	0.36
Q3	2.16	2.93	2.68	0.51	0.47	0.44
ICR	0.25	0.32	0.35	0.09	0.07	0.08

It can be observed that these beads are prepared using two concentration levels. There is a “high” concentration for Ce, Y, and In (∼2.5 fg/particle) and a low concentration for Tb, Bi, and Lu (∼0.5 fg/particle). In all cases, the mass distribution was relatively narrow, with RSD values below 15% that correspond to the dispersion in the mass distribution.

The arithmetic mean elemental mass was selected for subsequent response factor (α) calculations for each isotope in every sample after filtration of the bead data. In order to calculate isotopic response factors, the mean mass of each isotope was calculated considering the natural isotopic abundance of the bead-contained elements. Median, quartile, and interquartile range values are additionally reported in Table 1 to provide a more complete description of the experimental mass distributions but were not further used for calculations. Table 2 shows the average and dispersion (standard deviation) for the α values obtained independently for three samples. Mass-based isotopic response factors were converted into molar response factors by application of the atomic mass and natural isotopic abundance of each isotope. Once the response factors are obtained, they can be applied to convert the intensity of cell events into the mass of any of the isotopes contained in the beads.

**Table 2:** Response Factors Obtained for Each Natural Stable Isotope of the Bead-Contained Elements^a^

isotope	mass-based response factor (α) (DC/fg)	molar response factor (DC/fmol)
^89^Y	(3.06 ± 0.06)·10^2^	(2.72 ± 0.05)·10^4^
^113^In	(4.02 ± 0.09)·10^2^	(4.5 ± 0.1)·10^4^
^115^In	(4.31 ± 0.05)·10^2^	(4.96 ± 0.06)·10^4^
^140^Ce	(1.026 ± 0.004)·10^3^	(1.44 ± 0.01)·10^5^
^142^Ce	(1.02 ± 0.07)·10^3^	(1.4 ± 0.2)·10^5^
^159^Tb	(2.22 ± 0.03)·10^3^	(3.53 ± 0.06)·10^5^
^175^Lu	(2.50 ± 0.05)·10^3^	(4.37 ± 0.08)·10^5^
^176^Lu	(2.53 ± 0.07)·10^3^	(4.5 ± 0.1)·10^5^
^209^Bi	(1.32 ± 0.02)·10^3^	(2.75 ± 0.04)·10^5^

a Values are reported with a decimal precision determined by the corresponding standard deviation.

As a model, a B-cell chronic lymphocytic leukemia (MEC-I) cell line was used. These cells overexpress a transmembrane protein, CD20, that is normally expressed by mature B lymphocytes. To target this biomarker, a monoclonal anti-CD20 antibody was labeled with ^175^Lu, as detailed in Methods section. The mass of lutetium per cell is therefore proportional to the level of CD20 expression in the MEC-I cell line. The results of this quantification were compared with those obtained using the traditional external calibration with ionic standards. The calibration-based quantification strategy in CyTOF was recently proposed and validated in a previous publication.^24^ Therefore, the calibration by external standards serves as a reference method for validation. This comparison is shown in Figure 3 for three independent biological replicates (R1, R2, and R3) run on different days.

**Figure 3:**
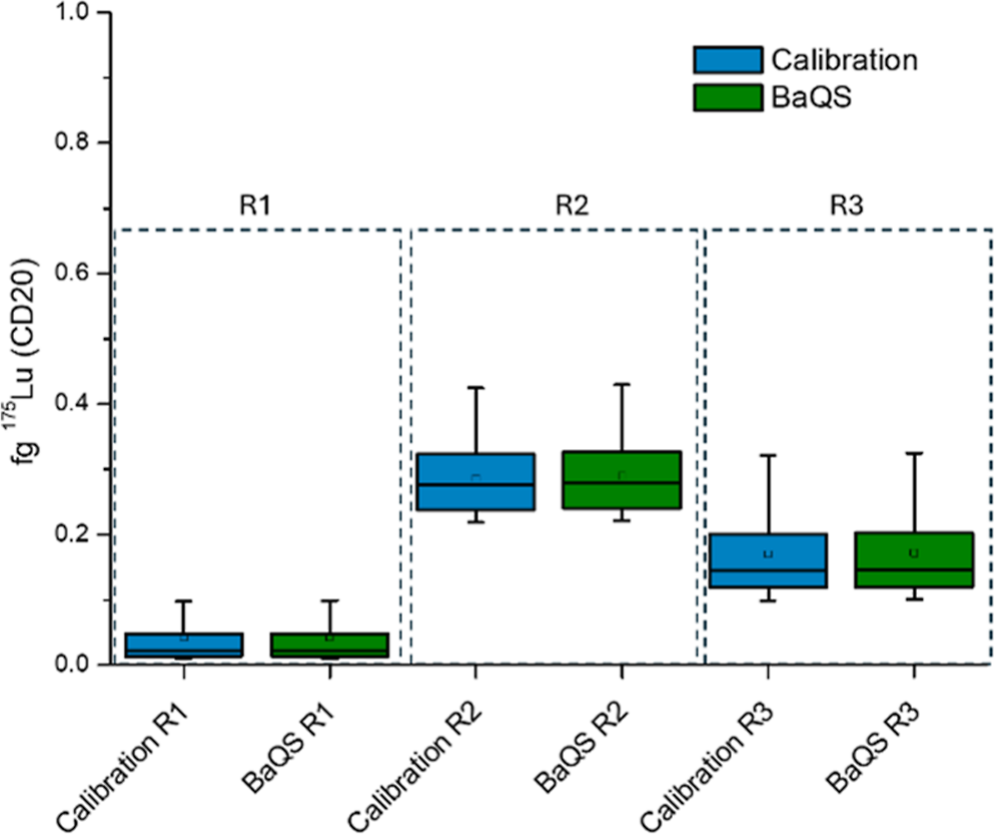
Comparison of the results obtained for ^175^Lu quantification in the MEC-I cell line expressing CD-20 after tagging de anti-CD 20 antibody with Lu by external calibration in CyTOF and BaQS strategy. ∼2000 cells were measured for every replicate (*n* = 3).

Statistical comparisons between quantification strategies were performed on the data in Figure 3 using biological replicates as the experimental unit. For each replicate, the median single-cell elemental mass was calculated, and differences between methods were assessed using a two-sided Mann–Whitney *U* test. Differences were considered statistically significant at *p* < 0.05. The results obtained by BaQS were not statistically different from those obtained by calibration in every replicate, which validates the proposed methodology. The differences among replicates reflect the biological variability of the data.

The BaQS method using the EQ-six beads is valid only for those six elements (eight isotopes) contained in the beads. However, if the user adds other types of beads, either commercially available or in-house-produced, this same strategy can be replicated for any element in the mass range from 75 to 209 that CyTOF instruments can monitor. Moreover, this strategy is not exclusively applicable to CyTOF instruments but also to any ICP–TOF–MS that allows the simultaneous measurement of several elements if adequate beads are added to the samples.

### BiRM: An Interpolated Model for Multielement Quantification

Taking advantage of the multielemental composition of the beads uniformly distributed along the CyTOF measuring range, their molar response factors can be estimated and plotted against the *m*/*z* of each of the isotopes contained on the beads. This is possible thanks to the high ionization efficiency achievable in the plasma for the elements of the beads that is close to 100%. In that case, previous publications demonstrated that the ICP–MS molar response can be modeled using different theoretical equations.^30,31^ Consequently, the molar responses (*n*_resp_) for all the 8 isotopes of the 6 elements contained in the beads (typically >5000 events) were calculated as in eq 3 and represented against the mass-to-charge ratio (*m*/*z*). The molar response, differently to the previously reported response factor (α_i_), takes into account the atomic mass of the element and isotope, the isotope abundance, and the concentration. This response was fitted empirically to polynomial functions of degree 2, 3, 4, and 5, resulting in the quartic function the one with the best correlation (*r* ^2^ = 0.99) while avoiding overfitting of the data. The response factors for all the isotopes are represented in Figure 4 (every data point considered for the fitting corresponds to the average intensity obtained for a minimum of 5000 bead events and the corresponding standard deviation), together with the fitted quartic polynomial function of eq 4. This behavior showing a mass-dependent response with a slight decrease at high *m*/*z* is also consistent with published results using ICP–TOF–MS instruments.^34^ The fourth-order polynomial was used as an empirical interpolation of the instrumental response within the calibrated mass range in our specific CyTOF instrument, without physical significance of the coefficients.

4 $$n_{resp}=-2.46\times 10^{-2}{\left(\frac{m}{z}\right)}^{4}+1.28\times 10^{1}{\left(\frac{m}{z}\right)}^{3}-2.36\times 10^{3}{\left(\frac{m}{z}\right)}^{2}+1.88\times 10^{5}\left(\frac{m}{z}\right)-5.44\times 10^{6}$$

**Figure 4:**
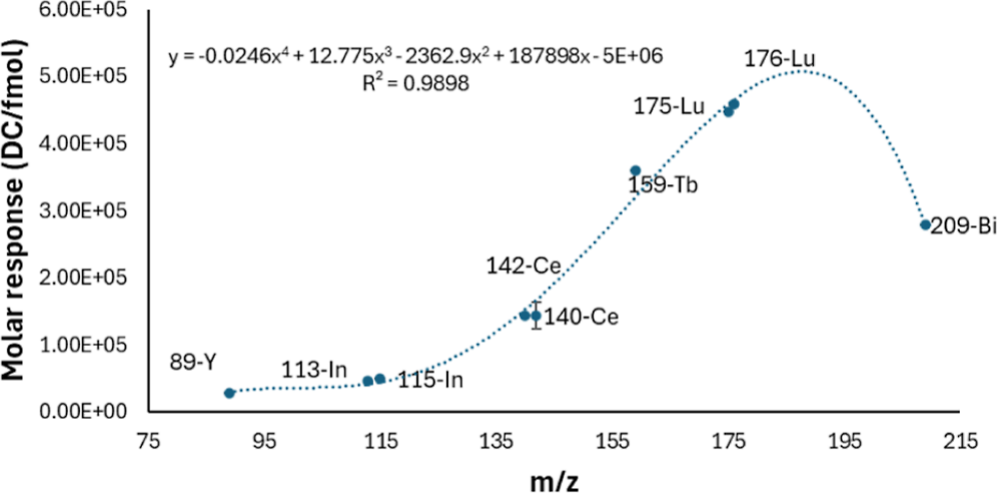
Molar response factors for every isotope in the beads as a function of *m*/*z* and the quartic function model (dashed line) that fits such data obtained using CyTOF results. Standard deviation of each data point is also included (only distinguishable in the case of ^140^Ce).

Using the *m*/*z* of the isotope of interest and by interpolation in the curve, it is possible to estimate its molar response on each sample (matrix matched). Out of the obtained molar response and taking the integrated dual counts obtained for such isotope in the sample, inferring its mass in a single cell event is straightforward. Figure 5A shows the correlation between the predicted (using eq 4) and the experimental value (using eq 3) for the molar responses of the isotopes contained in the beads. A good correlation can be observed (*R* ^2^ = 0.99), with a slope of 0.99, suggesting an excellent ability of the model to calculate the molar sensitivity of these isotopes.

**Figure 5:**
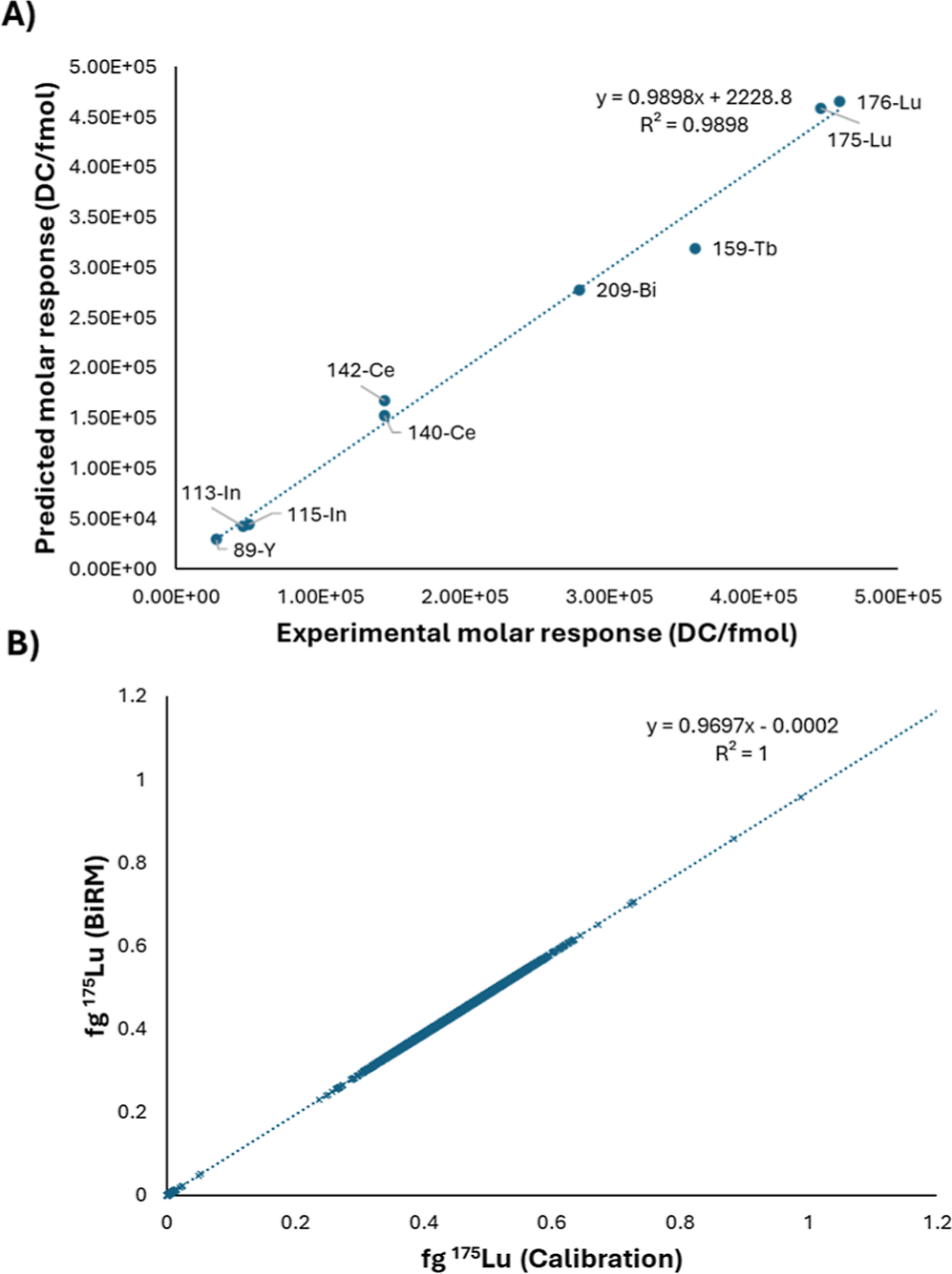
(A) Plot showing the correlation between the BiRM-predicted and the experimental molar response for the multielemental beads and (B) the correlation of 8200 individual cell events obtained by BiRM versus calibration with ^175^Lu standards. Error bars were omitted for clarity. Experimental uncertainties associated with the molar response factors are reported in Table 2.

Although it is possible to perform the described calculations using a datasheet like from microsoft excel, Google Sheets, or LibreOffice Calc, the BiRM interpolation and quantification workflow becomes increasingly cumbersome as the number of isotopes, events and biological replicates grows. For this reason, an in-house script has been developed in R, which is provided as Supporting Information. The script, which is fully transparent and open-access, operates directly on the data matrix generated for either cell events or calibration-bead events after standard CyTOF preprocessing (see the Experimental section). By introducing the elemental information required for BiRM calculations as described in the accompanying “Readme” file, the script automatically computes the molar or mass amount of each element per cell. In addition, it generates summary statistics, diagnostic figures and histograms for rapid quality assessment. This implementation ensures reproducibility, minimizes operator-dependent variability and allows users to reprocess previously acquired data sets without any additional experimental steps. A continuously updated version of software will be made available in a public repository, where future improvements, extension and documentation will be deposited. Because the isotopes used in Figure 5A were also employed to construct the BiRM model, an additional leave-one-out validation was performed to assess its predictive capability. In each iteration, one calibration isotope was excluded from the fitting procedure and its molar response factor was subsequently predicted using a model constructed from the remaining isotopes. The predicted values were then compared with the experimentally determined molar response factors. The leave-one-out validation yielded a mean absolute relative error (MARE) of 14.3%, compared with 8.3% for the all-in model. As expected, prediction errors increased when an isotope was excluded from the model construction. However, the obtained deviations remained within a reasonable range considering the limited number of calibration points available for model fitting.

Larger deviations were observed for isotopes located at the boundaries of the calibrated mass range (^89^Y and ^209^Bi), whose exclusion transforms the prediction into an extrapolation rather than an interpolation problem due to the absence of neighboring calibration points on one side of the fitted mass range. Consequently, these two isotopes were excluded from the MARE calculations for both the all-in and the leave-one-out models. The complete leave-one-out validation results are provided in Table S1.

To test the suitability of this quantification strategy, the methodology was applied to the quantification of Lu in the same cell model as the one used for BaQS: MEC-I labeled with the ^175^Lu-tagged anti-CD20 antibody. The results obtained for ^175^Lu quantification by BiRM were not statistically different from those obtained by BaQS and with the external calibration curve, as can be seen in Figure 5B. There is a total correlation between the results obtained by external calibration and BiRM for ∼8200 cell events with a slope of 0.97, which indicates that the bead-contained lutetium works as well as a standard (BaQS) as when its response is predicted by BiRM.

In order to extend this quantification strategy to a higher number of biomarkers, the cell line was now shifted to ovarian carcinoma A2780, with a higher availability of lanthanide-labeled antibodies against positively expressed biomarkers. In this case, the cells are known to overexpress the transferrin receptor 1 (TfR1) and also an ovarian cancer-specific biomarker, ErbB2 receptor (HER2). The corresponding monoclonal antibodies anti-TfR1 and anti-HER2 were labeled with ^142^Nd and ^175^Lu, respectively. Additionally, the iridium DNA intercalator that is added as a cell marker to every sample in CyTOF was also quantified. The main advantage of the BiRM strategy is that it can also be applied to estimate the molar response and, therefore, the mass of any other element within the mass range that is not necessarily present in the beads. In this newly labeled cell line, the BiRM strategy was now used not only to quantify ^175^Lu (HER2), but also ^142^Nd (TfR1) and ^193^Ir (DNA intercalator) that are not present in the beads. The predicted molar response factors obtained by the BiRM are shown in Table 3. For comparison, the molar response factors obtained by calibration using external standards are shown in the third column of Table 3.

**Table 3:** Predicted Molar Response Factors Obtained by BiRM and Those Obtained by Calibration for Elements Not Present in the Multielemental Beads

isotope	molar response factor (BiRM) (DC/fmol)·× 105	molar response factor (calibration) (DC/fmol) × 105
^175^Lu	4.5 ± 0.1	4.3 ± 0.1
^142^Nd	1.81 ± 0.01	1.56 ± 0.02
^193^Ir	4.7 ± 0.1	4.9 ± 0.1

In all three isotopes, the error between the molar response factors obtained by calibration and BiRM was between 4 and 20%, which might be ascribed to miscalculation of the transport efficiencies, required to adjust the transport of inorganic standard. It is noteworthy that the BiRM strategy does not require accounting for element TE because single-particle signal intensities are applied to single-cell events. The BiRM quantification strategy avoids not only TE calculation and the associated errors assuming fixed particle concentrations or sizes but also the assumption that the standard gold nanoparticles behave exactly as the ionic standards.

Quantification either by calibration, BaQS or BiRM strategies allow to calculate the mass of lanthanide isotope per cell (Figure S1). In this study, the anti-HER2 and anti-TfR1 antibodies were characterized in terms of lanthanide atoms per antibody (detailed in the Methods section), obtaining a stoichiometry of 75 ± 16 atoms of ^175^Lu per anti-HER2 molecule and 146 ± 8 atoms of ^142^Nd per anti-TfR1 molecule. This allows to calculate the number of HER2 and TfR1 per cell, as shown in Figure 6A,B. The results in Figure 6A show the excellent agreement between the results obtained for lutetium using the external standards calibration, the experimental molar response (BaQS), and the predicted molar response (BiRM) for three independent biological replicates. Figure 6B,C shows the other two quantified isotopes, ^142^Nd, as number of TfR1 molecules per cell, and ^193^Ir, respectively. In these cases, the BaQS strategy is not possible because these two elements are not included in the beads. ^193^Ir intercalates DNA and there is no biological individual entity to quantify. Therefore, it appears in Figure 6C as mass of ^193^Ir per cell. As can be observed, no significant differences were detected between the calibration and BiRM quantification strategies in any replicate for any element, revealing the excellent correlation between the traditional calibration-based quantification strategy and the BiRM, even applied to elements that are not contained in the beads.

**Figure 6:**
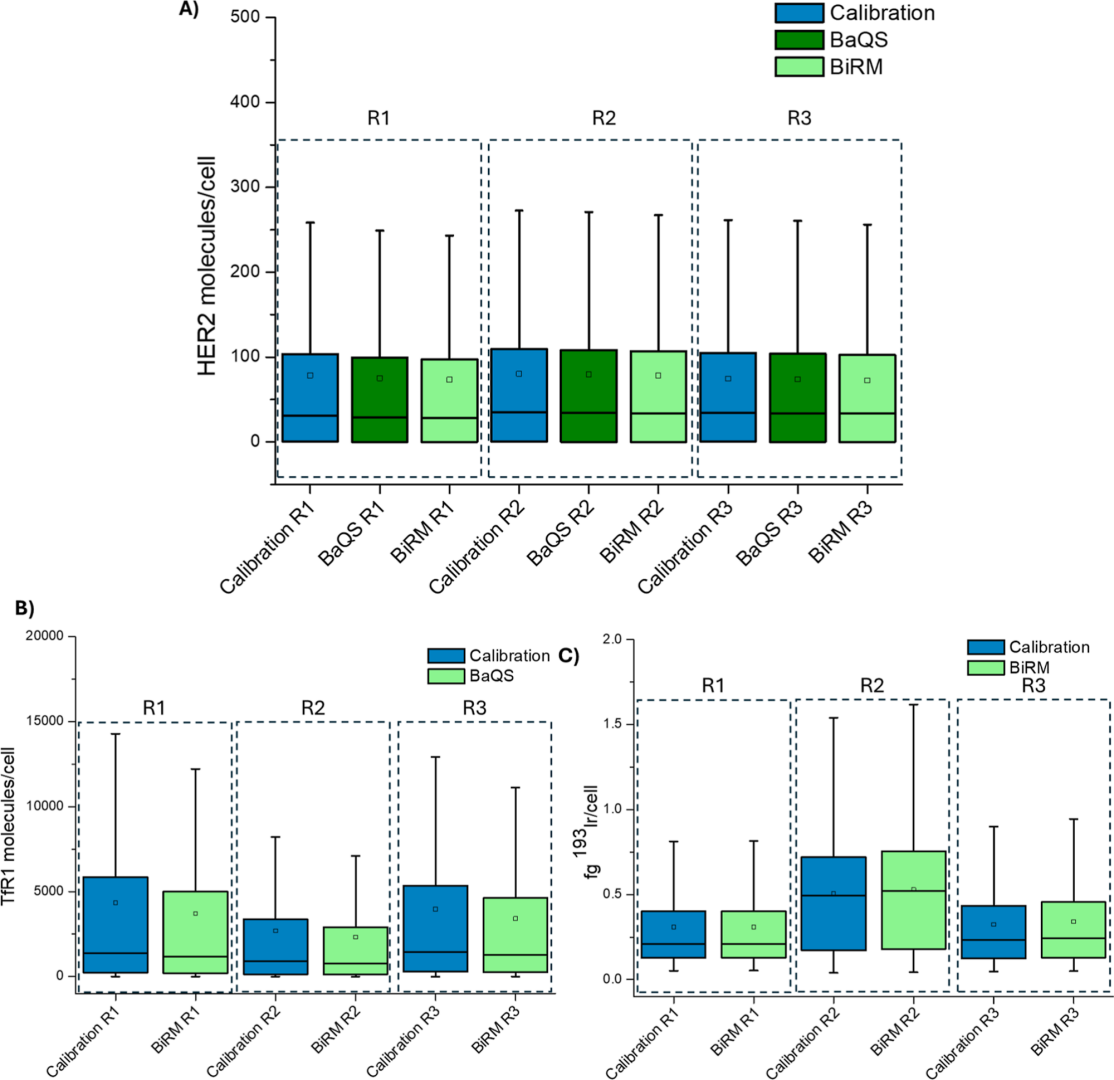
Whiskers and box plot showing the results for three independent biological replicates of A2780 cells labeled with anti-HER2 tagged with ^175^Lu (A), anti-TfR1 tagged with ^142^Nd (B), and the DNA intercalator containing ^193^Ir (C). Comparison of the results obtained by external calibration, BaQS (only for A) and BiRM strategies. Results on HER2 (A) and TfR1 (B) are expressed as molecules per cell. Results on the iridium intercalator (C) are expressed as mass of Ir per cell. More than 30,000 cells were acquired for every replicate.

Compared with traditional calibration strategies in CyTOF or SC–ICP–MS, typically relying on external ionic standards and assumptions about TE, both BaQS and BiRM approaches offer an internally referenced quantification framework. In terms of repeatability, BaQS and BiRM provide highly consistent within-run quantification because the bead events are acquired concurrently with the cells in every sample, stabilizing the response factor for the isotopes present in the beads sample-to-sample (see Figure S2). BiRM even extends this advantage by modeling the mass-dependent response across the full *m*/*z* range, providing repeatable estimates even for isotopes that are not contained in the beads. Regarding robustness, both BaQS and BiRM correct for short- and long-term fluctuations in the element response because the calibration standard is present in every acquisition, whereas the external calibration approaches remain affected by changes in the sensitivity and TE during the acquisition of the samples as well as matrix-derived effects. Regarding the applicability, BaQS is naturally limited to the isotopes present in the beads, although the production of characterized beads with different metal contents would allow an increase in the number of analytes. However, BiRM is broadly applicable, provided the mass range is fully calibrated in this case. The current implementation of BiRM is expected to be most reliable for elements whose first ionization potentials are comparable to those of the calibration elements used to construct the model (Y, In, Ce, Tb, Lu, and Bi, approximately 5.4–7.3 eV). Elements within this range are expected to exhibit similar ionization behavior in the ICP close to 100% and therefore follow the empirical response trend described by the model. For elements with substantially higher ionization potential, additional validation may be required before quantitative application.

Both BiRM and BaQS eliminate the need for repeated external standard curves, transport-efficiency measurements, and standards, thereby decreasing sample preparation time, instrument use, and experimental complexity. It is worth mentioning, however, that specially BiRM applied to nonbead-contained elements, but also BaQS, provide estimations of the response factors, which may decrease the accuracy of these methods. On the other hand, the developed quantification strategies do not depend on TE determination, which also reduces the number of sources of inaccuracy. It must be noted that both BaQS and BiRM can be applied not only to CyTOF-based analyses but also to any SC–ICP–MS instrument with multielemental capabilities. Currently, this includes basically instruments with TOF mass analyzers (ICP–TOF–MS).

## Conclusion

This study presents a practical route to quantitative mass cytometry by repurposing the multielement calibration beads that are routinely added to CyTOF samples as in-tube quantitative standards that can be extended not only to CyTOF but also to any other TOF-based ICP–MS instruments. Two complementary strategies were proposed. First, BaQS performs single-point calibration for bead-encoded isotopes to obtain the absolute mass of lanthanide per cell. Second, the BiRM makes use of the multielement bead signature to empirically model the instrumental response across the monitored *m*/*z* window, enabling the prediction of response factors for isotopes that are not present in the beads. The results indicate that both strategies provided accurate results with no statistically significant differences with the validated external standards calibration for the quantification of bead-contained and noncontained lanthanides, being BaQS and BiRM simpler, faster, more efficient, and ready to be applied to already acquired data with no additional measurements needed.

## Supplementary Material





## Notes

Single-cell elemental mass distributions and signal stability for EQ Six Element Beads (PDF)
Model of the R script used for data processing (ZIP)
