# Supplementary material for: Quantitative Single-Cell Multielemental Analysis Using Metal-Loaded Microparticles: toward a Universal Quantification Workflow in Mass Cytometry
Source: Anal Chem. 2026 Jul 24;98(31):22966–76. doi: 10.1021/acs.analchem.6c02340 (PMC13470980; doi:10.1021/acs.analchem.6c02340)
Supplement: Supplementary file 1 [file ac6c02340_si_001.pdf]

Supporting Information

Quantitative single-cell multielemental analysis using metal-loaded microparticles: towards a universal quantification workflow in mass cytometry

Ángela de la Rosa-Díaz[1,2], Carlos López-Portugués[1,2], Mario Corte-Rodríguez[1,2,*], María Montes-Bayón[1,2,*].

[1] Department of Physical and Analytical Chemistry, University of Oviedo. Julián Clavería 8, 33006 Oviedo, Spain.

[2] Health Research Institute of Asturias (ISPA). Av. De Roma s/n, 33011 Oviedo, Spain.

E-mail: cortemario@uniovi.es, montesmaria@uniovi.es

Supplementary Figures

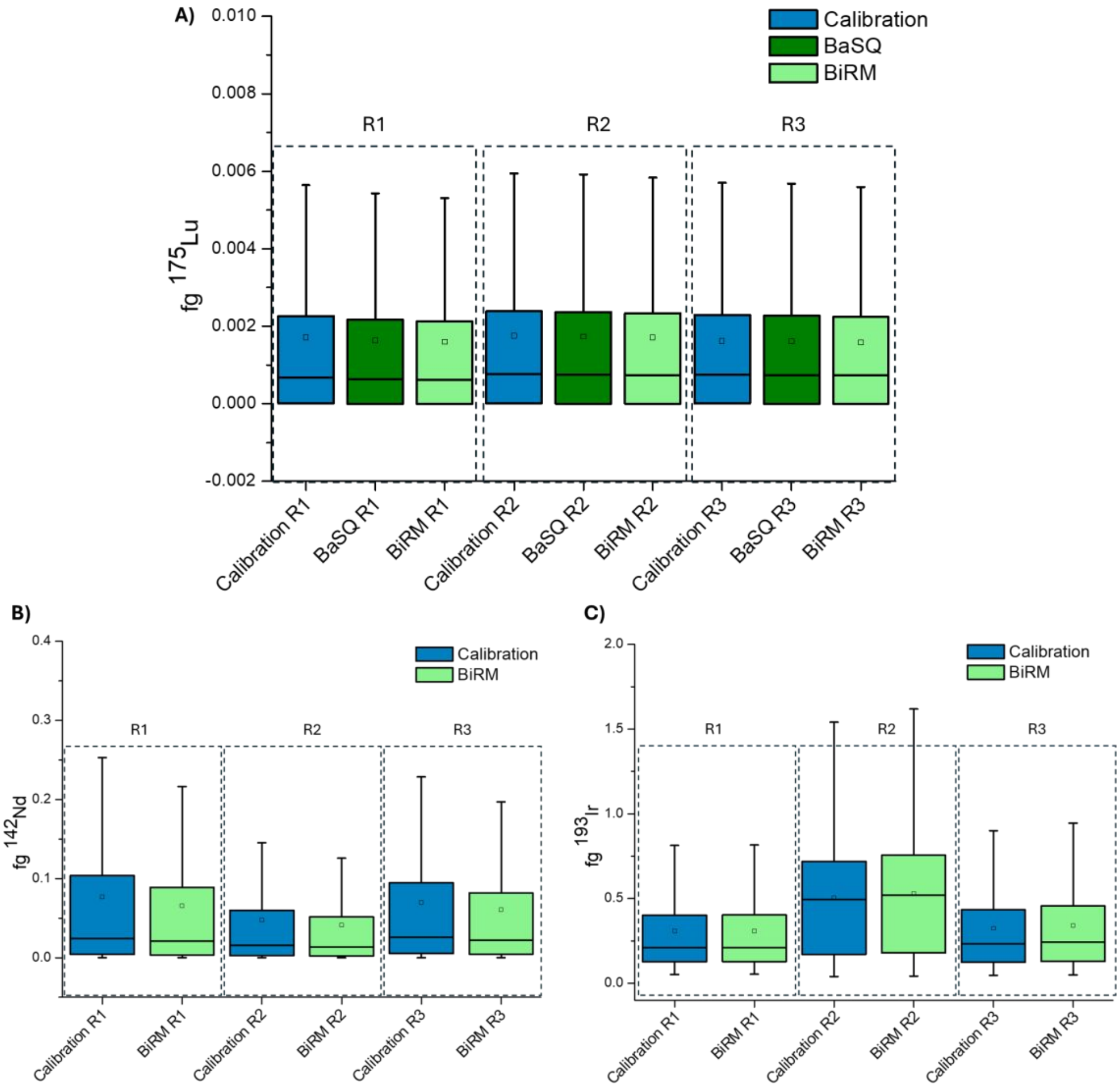

Figure S1: Whiskers and box plot showing the results of the mass of the labelling element for three independent biological replicates of A2780 cells labelled with anti-HER2 tagged with ^{175}Lu (A), anti-TfR1 tagged with ^{142}Nd (B), and the DNA intercalator containing ^{193}Ir (C). Comparison of the results obtained by external calibration, BaQS (only for A) and BiRM strategies.

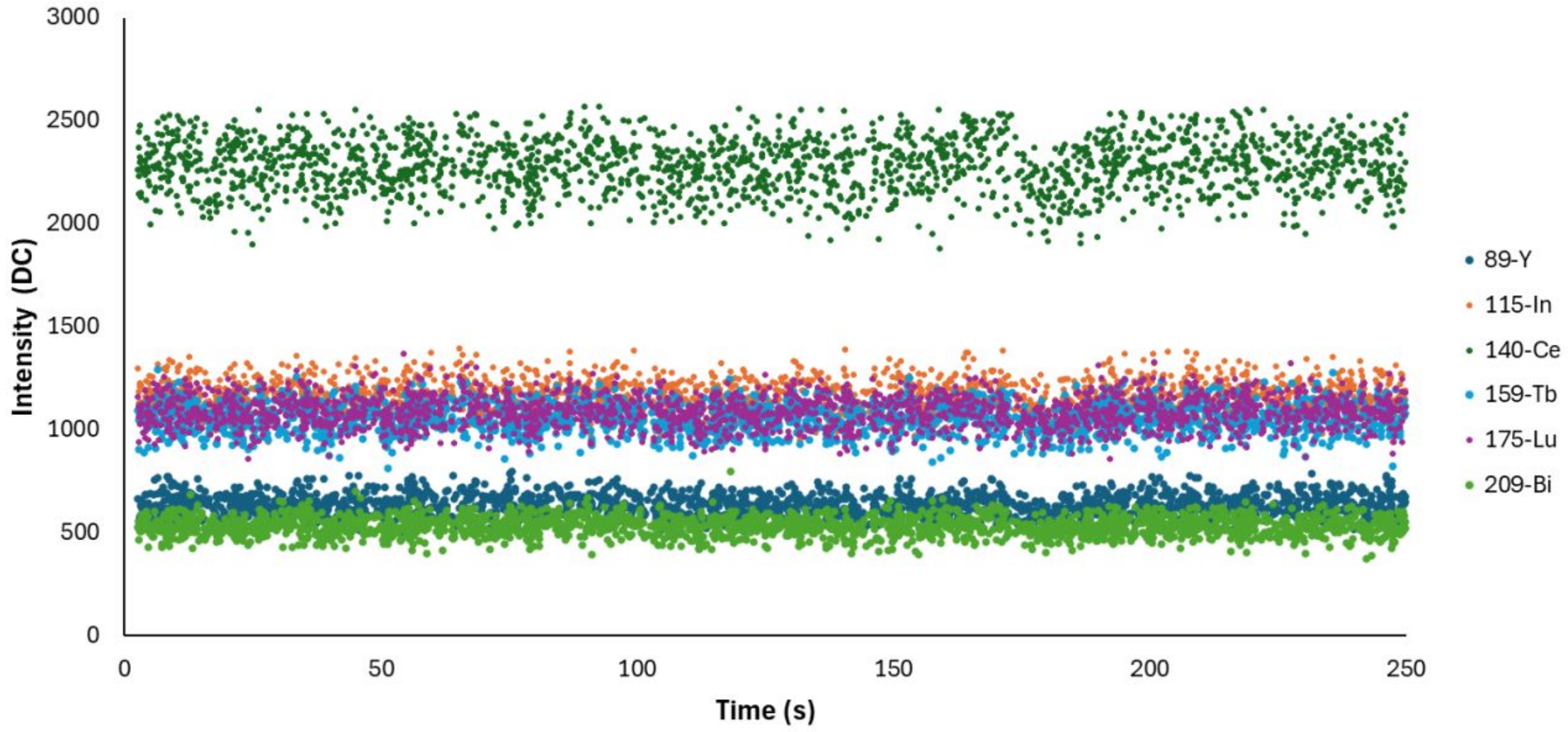

Figure S2: Signal variation within a 250 seconds measuring time for the EQ™ Six Calibration Beads. Each dot corresponds to a single bead detected at a certain time point. All 6 elements are monitored in every single bead via their most abundant natural isotopes where several isotopes exist.

Supplementary Tables

Table S1. Molar responses calculated with the full model and the leave-one-out validation, leaving one isotope out of the model and predicting its molar response. Relative errors are calculated with respect to the experimental molar response.

Isotope out	Experimental response	All-in predicted response	Leave-one-out predicted response	All-in relative error (%)	Leave-one-out relative error (%)
89	2.78E+04	2.97E+04	9.53E+05	6.8	3326.3
113	4.66E+04	4.20E+04	3.73E+04	-9.8	-19.9
115	5.03E+04	4.47E+04	3.98E+04	-11.1	-20.8
140	1.44E+05	1.53E+05	1.58E+05	5.8	9.5
142	1.44E+05	1.67E+05	1.83E+05	15.9	26.5
159	3.59E+05	3.19E+05	3.01E+05	-11.1	-16.2
175	4.47E+05	4.59E+05	4.69E+05	2.7	4.9
176	4.60E+05	4.66E+05	4.71E+05	1.3	2.6
209	2.79E+05	2.78E+05	-1.23E+06	-0.5	-539.5
