# Supplementary material for: Quantitative Single-Cell Multielemental Analysis Using Metal-Loaded Microparticles: toward a Universal Quantification Workflow in Mass Cytometry
Source: Anal Chem. 2026 Jul 24;98(31):22966–76. doi: 10.1021/acs.analchem.6c02340 (PMC13470980; doi:10.1021/acs.analchem.6c02340)
Supplement: Supplementary file 2 [file ac6c02340_si_002.zip › SupportingInformation_2/BiRM_Quantification_README.docx]

# README – EQ 6 Element Beads Quantification Script

**Overview**

This R script performs quantitative elemental analysis at the single-cell level using EQ 6 Calibration Beads as standards in the so-coined Bead-inferred Response Model (BiRM), specially designed for CyTOF data.

It converts detector counts (DC) into femtograms of element per cell using a bead-derived molar response curve.

The script:

- Builds a 4th-degree molar response calibration curve from EQ beads

- Calculates the molar sensitivity in DC/fmol for a selected isotope

- Converts cell event signals into fmol and femtograms per cell

- Displays the molar response curve and histogram

- Exports a CSV file with per-cell element mass

- Shows mean ± standard deviation in console and popup

**Requirements**

- R (version ≥ 4.0 recommended)

- RStudio

- Package: ggplot2 (installed automatically)

**Input Files**

Two CSV files must be in the same folder as the script:

1) Beads events file

2) Cell events file

Both CSV files must contain the events matrix exported by FlowJo or other .fcs compatible software after adequate data cleanup and gating.

**User Configuration**

Edit only the section separated with “== Lines”:

GENERIC PART: define ONE element to quantify

**Parameters:**

- element_label: name used in outputs

- isotope_col: column name in cells CSV

- isotope_mass: isotopic mass

- isotope_abundance: fraction (not percent)

- element_atomic_mass: atomic mass (g/mol)

**Running the Script**

Open the script in RStudio and click Source or press Ctrl + Shift + Enter.

Do not use 'Source as Background Job'.

**Outputs**

- Molar response curve plot

- Histogram of element mass per cell

- Popup with mean ± SD

- CSV file with per-cell values

The output CSV contains one row per cell with femtograms per cell.
